# *miR-1-3p* Downregulation as a Consistent Biomarker for Atrial Fibrillation Burden in Patients with Sick Sinus Syndrome: A Multi-Sample Analysis

**DOI:** 10.3390/ijms26104936

**Published:** 2025-05-21

**Authors:** Hui-Ting Wang, Shyh-Ming Chen, Huang-Chung Chen, Pei-Ting Lin, Yung-Lung Chen

**Affiliations:** 1 Emergency Department, Kaohsiung Chang Gung Memorial Hospital, Kaohsiung 833, Taiwan; gardinea1983@gmail.com; 2 School of Medicine, College of Medicine, National SunYat-sen University, Kaohsiung 80424, Taiwan; 3 Division of Cardiology, Department of Internal Medicine, Kaohsiung Chang Gung Memorial Hospital, Chang Gung University College of Medicine, Kaohsiung 833, Taiwan; syming99@yahoo.com.tw (S.-M.C.); chc3@cgmh.org.tw (H.-C.C.); r40391132@gmail.com (P.-T.L.); 4 Graduate Institute of Clinical Medical Sciences, College of Medicine, Chang Gung University, Taoyuan 333, Taiwan

**Keywords:** atrial fibrillation, miRNA, *miR-1-3p*

## Abstract

Atrial fibrillation (AF) is a leading cause of stroke, heart failure, and cardiovascular morbidity, yet its pathophysiology remains incompletely understood. Among various molecular regulators, microRNAs (miRNAs) have emerged as promising biomarkers for AF detection and burden monitoring. However, the optimal sample type for miRNA analysis remains unclear, posing a challenge for biomarker standardization. This study aimed to assess whether miRNA expression profiles remain consistent across plasma and blood cells, with a focus on identifying miRNAs with a strong predictive potential for AF burden. This exploratory study recruited patients diagnosed with sick sinus syndrome who had undergone permanent pacemaker implantation. Participants were stratified into three groups based on AF status: no AF (*n* = 2), paroxysmal AF (PaAF; *n* = 2), and persistent AF (PerAF; *n* = 2) for white blood cell (WBC) samples, and pooled plasma samples from no AF (*n* = 3 pools) and PerAF (*n* = 3 pools). Using an miRNA microarray analysis, *miR-1-3p* was consistently downregulated in both WBC and plasma samples of patients with AF, showing significant decreases (fold-change in WBC: PaAF 0.22, PerAF 0.20; plasma PerAF 0.28) and highlighting its potential as a circulating biomarker for AF burden. Additional differentially expressed miRNAs, including *miR-451a* and *miR-382-5p*, exhibited sample-dependent variations, underscoring the importance of validating miRNA expression across multiple biological compartments. The study highlights the need for mechanistic investigations to determine whether *miR-1-3p* directly contributes to AF pathogenesis or serves as a downstream consequence of atrial remodeling. These findings reinforce the potential of *miR-1-3p* as a reliable circulating biomarker for AF, offering new avenues for non-invasive monitoring and risk stratification. Future research should explore the role of *miR-1-3p* in AF-related molecular pathways and its applicability as a therapeutic target.

## 1. Introduction

Atrial fibrillation (AF) is the most common sustained cardiac arrhythmia, contributing significantly to stroke, heart failure, and cardiovascular morbidity [1]. Despite advancements in AF management, its pathophysiology remains incompletely understood, with inflammation, fibrosis, and electrical remodeling being key contributors to disease progression.

Paroxysmal AF (PaAF) frequently precedes the development of persistent AF (PerAF). Studies have shown that patients with PerAF have a higher risk of mortality and thromboembolism compared with those with PaAF [2]. Therefore, identifying the risk of progression in patients with PaAF is clinically significant. However, while some methods have been explored [3], no widely accepted methods currently exist to predict the transition from PaAF to PerAF. Identifying reliable biomarkers for AF diagnosis, risk stratification, and therapeutic targeting remain major challenges in clinical research.

MicroRNAs (miRNAs), a class of small non-coding RNAs that regulate gene expression at the post-transcriptional level, have emerged as potential biomarkers for cardiovascular diseases. Numerous studies have demonstrated that specific miRNAs are involved in AF-related structural and electrical remodeling, particularly those associated with inflammatory and fibrotic pathways [4,5,6]. *miR-1* and *miR-133* are significantly upregulated in AF atrial tissue, affecting ion channel function and electrical remodeling [7]. *miR-21* and *miR-29b* contribute to atrial fibrosis by regulating collagen synthesis and extracellular matrix deposition [8]. *miR-146a* is involved in inflammation and structural remodeling, with elevated levels in AF atrial tissue and plasma [9].

Currently, plasma is the predominant biological source used for circulating miRNA biomarker research in AF. A systematic review focusing on circulating miRNAs in AF revealed that among the included 12 studies, 8 utilized plasma as the primary sample type, while only a few studies analyzed miRNAs derived from serum or platelet-enriched fractions, which strongly recommended extracting cell-free miRNAs from plasma using a stringent double-centrifugation protocol to minimize contamination from residual cellular components and ensure the reproducibility and reliability of the quantitative results [10]. Biological sources, such as serum or platelet-enriched samples, for miRNA detection in AF research. The lack of consistency in sample type selection and pre-analytical processing methods across these studies may partly explain the discrepancies observed in miRNA biomarker outcomes [11]. This variability underscores the scientific establishment of standardized protocols for sample preparation and miRNA quantification, aiming to ensure methodological consistency and reproducibility across research laboratories.

Additionally, practical considerations, such as ease of processing and specimen availability, also influence clinical applicability. Identifying miRNAs with consistent expression patterns across different biological samples would substantially enhance the feasibility and practicality of circulating miRNA biomarker studies, facilitating their broader adoption in both future research and clinical practice.

This study aims to investigate whether miRNA expression patterns remain consistent across different sample sources (plasma and white blood cells (WBCs)) and to determine the feasibility of using WBCs as an alternative and potentially more practical source for AF biomarker screening. Given that long-term sample storage (e.g., −80 °C for 5 to 10 years) poses logistical and financial challenges, optimizing a single robust sample type for the miRNA analysis could enhance the clinical applicability of such biomarkers.

Furthermore, given the high cost of large-scale miRNA profiling—where analyzing 100 miRNAs per patient can amount to substantial financial burdens—the study employs a two-phase approach: an initial screening phase followed by validation. This stepwise strategy allows for the identification of candidate miRNAs associated with AF-related inflammation and fibrosis before expanding to larger cohorts for further validation. This methodology is critical for balancing cost-efficiency with scientific rigor in biomarker discovery.

## 2. Results

### 2.1. Differential miRNA Expression in WBC in Patients with PerAF, PaAF, and No AF

**Figure 1:**
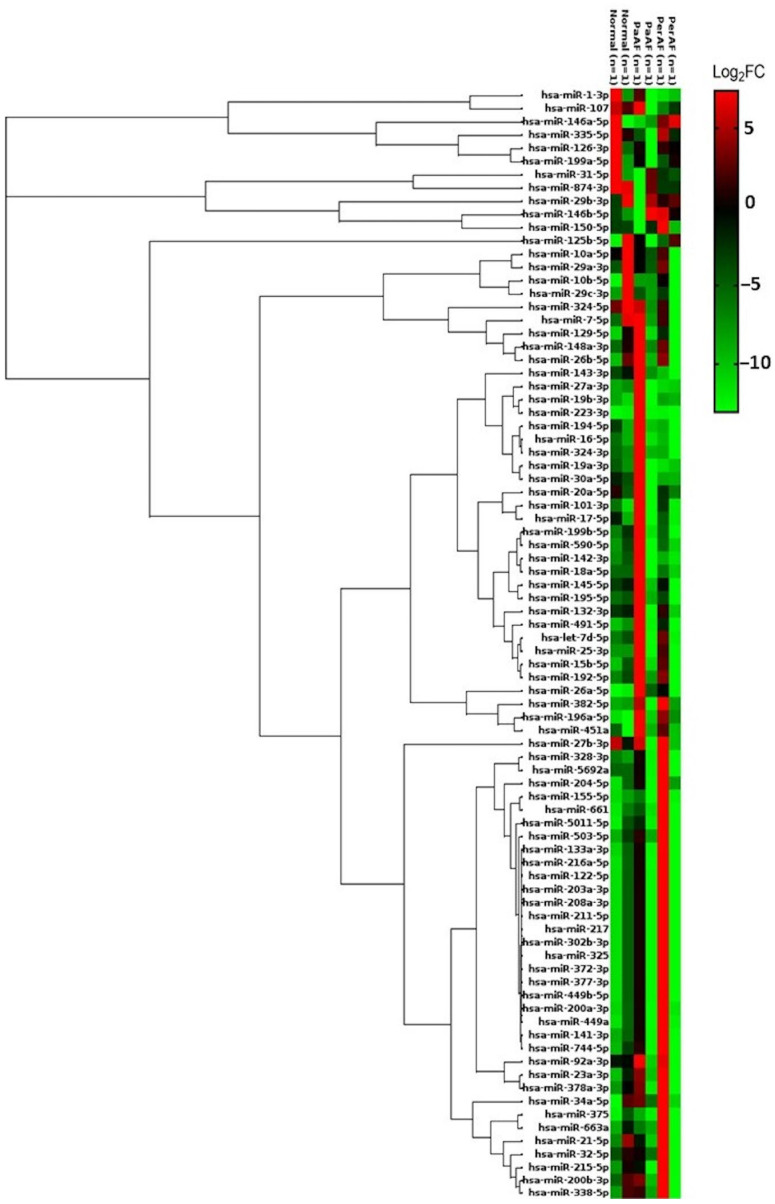
Heatmap of differentially expressed miRNAs in WBCs from patients with AF (PaAF and PerAF) versus no-AF controls. We identified 9 significant miRNAs in PaAF and 12 in PerAF (fold-change ≥1.5 or ≤0.5, or *p* < 0.05), with 5 overlapping; the heatmap depicts the 16 unique WBC miRNAs dysregulated in at least one AF group. Samples and miRNAs are organized by unsupervised hierarchical clustering, revealing distinct expression patterns by group. Red denotes higher expression and green denotes lower expression relative to the control group. The clustering shows that patients with AF segregate from controls based on WBC miRNA profiles. PaAF and PerAF share several dysregulated miRNAs but also exhibit specific trends—for example, *miR-1-3p* is consistently downregulated in both AF types, whereas *miR-223-3p* is upregulated in PaAF but reduced in PerAF. Such patterns indicate that WBC miRNA dysregulation parallels AF severity, with certain miRNAs potentially marking the progression from paroxysmal to PerAF.

To investigate the potential role of miRNAs in AF burden, we analyzed miRNA expression profiles in WBCs from patients with sick sinus syndrome who had received pacemaker implantation. The cohort was stratified into three groups: patients without AF (No AF), those with PaAF, and those with PerAF. A fibrosis miRNA microarray analyzing 84 miRNAs was performed. A differential expression analysis identified 9 significantly altered miRNAs in the PaAF group (fold change ≥1.5 or ≤0.5, or *p* < 0.05) and 12 miRNAs in the PerAF group, with 5 miRNAs shared between both the AF groups (Figure 1).

Among the five common miRNAs, miR-451a and miR-196a-5p were consistently upregulated in both PaAF and PerAF, whereas miR-1-3p was significantly downregulated across both AF subtypes. miR-125b-5p exhibited a bidirectional trend, showing downregulation in PaAF but upregulation in PerAF. miR-223-3p demonstrated an inverse expression pattern, being increased in PaAF but decreased in PerAF.

### 2.2. Plasma miRNA Profiling in Patients with PerAF and No AF

**Figure 2:**
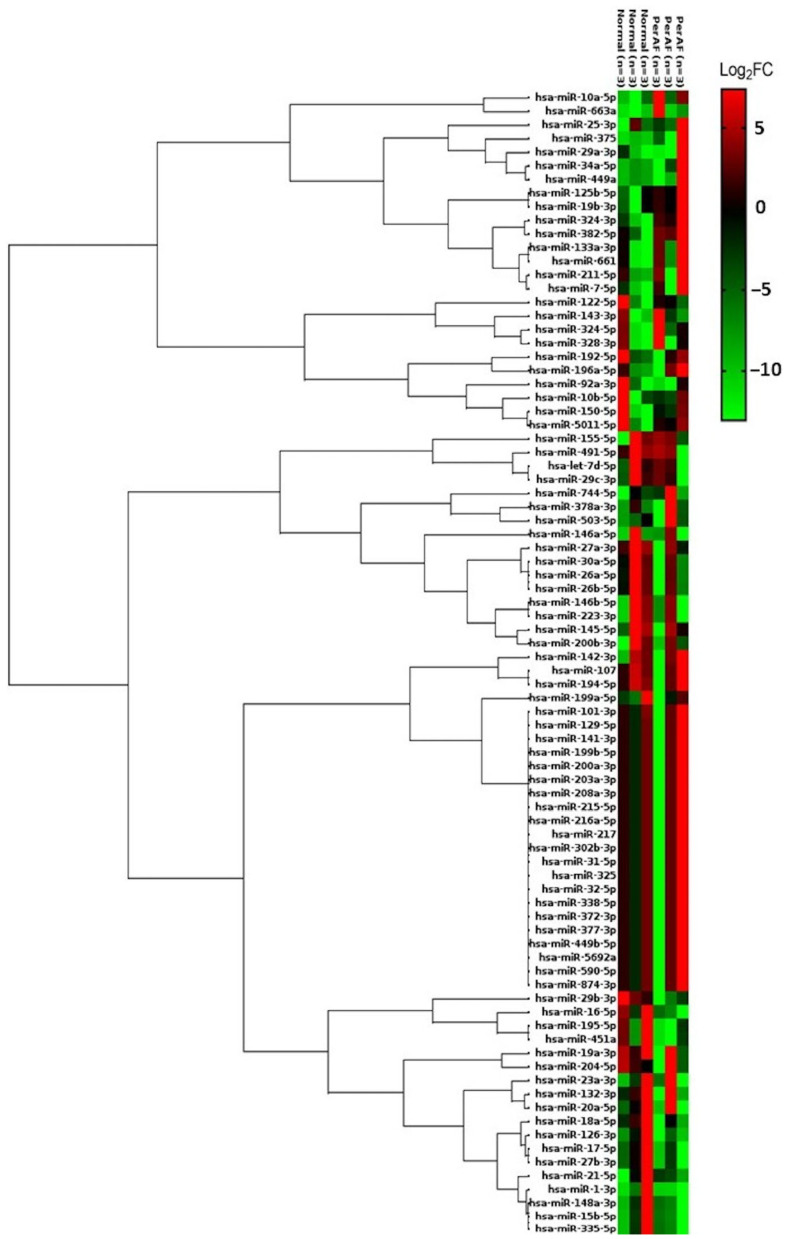
Heatmap depicting miRNA expression profiles in plasma samples from patients with PerAF compared with non-AF controls. We identified 47 significantly dysregulated miRNAs in the PerAF group compared with the control group (fold-change ≥1.5 or ≤0.5, or *p* < 0.05). The dendrogram reflects unsupervised hierarchical clustering of both samples and miRNAs, illustrating grouping by AF status. Expression levels are represented by a red–green color scale: red indicates upregulation (higher expression) and green indicates downregulation (lower expression) relative to the control group. Notably, patients with AF display a broad pattern of miRNA dysregulation in plasma, suggesting that significant circulating miRNA alterations emerge with disease progression.

Am analysis of plasma miRNA expression was conducted in No AF and PerAF groups to further elucidate the systemic miRNA alterations associated with AF. A total of 9 miRNAs were upregulated, whereas 38 miRNAs were downregulated in the PerAF group compared with the No AF group (Figure 2). Among these, miR-16-5p, miR-29b-3p, miR-324-3p, and miR-382-5p exhibited significant differential expression (*p* < 0.05), highlighting their potential as circulating biomarkers for AF detection [12,13,14].

### 2.3. Cross-Compartment Comparisons of WBC and Plasma miRNA Expression

To identify consistent miRNA expression patterns across different biological compartments, we compared miRNA profiles in WBCs and plasma samples from patients in the No AF and PerAF groups. Five miRNAs demonstrated consistent expression changes between the two sample types (Table 1). *miR-382-5p* was upregulated in both WBC and plasma, whereas *miR-338-5p* and *miR-145-5p* were consistently downregulated. In contrast, *miR-143-3p* and *miR-204-5p* exhibited divergent trends between WBCs and plasma, suggesting tissue-specific regulatory mechanisms.

### 2.4. miR-1-3p as a Potential Biomarker for AF Burden

Among the identified miRNAs, *miR-1-3p* exhibited a robust and consistent downregulation across all the comparisons. In WBCs, *miR-1-3p* was significantly reduced in both the PaAF and PerAF groups, with a similar decline observed in plasma samples from patients with PerAF. This suggests that *miR-1-3p* may serve as a reliable biomarker that is indicative of AF progression and burden (Table 2).

## 3. Discussion

This study demonstrated the pivotal role of miRNAs in the progression of AF and their potential as biomarkers. By profiling miRNA expression in both WBCs and plasma, we observed distinct miRNA changes associated with AF severity. Notably, patients with PaAF and PerAF exhibited significant alterations in WBC miRNAs, with five miRNAs (*miR-223-3p*, *miR-451a*, *miR-196a-5p*, *miR-125b-5p*, and *miR-1-3p*) commonly dysregulated in both AF forms. In plasma, patients with PerAF (versus those without AF) showed broad miRNA perturbations (9 upregulated and 38 downregulated, with 5 reaching statistical significance: *miR10a-5p, miR-16-5p, miR-29b-3p, miR-324-3p, miR-382-5p*). Comparing WBC and plasma profiles revealed seven miRNAs found in both compartments; for example, *miR-382-5p* was consistently elevated, whereas *miR-129-5p*, *miR-338-5p*, and *miR-145-5p* were uniformly reduced in patients with AF. Among those miRNAs in our study, *miR-1-3p* was decreased across all the AF groups in both WBCs and plasma. Our results indicate that *miR-1-3p* is decreased in both WBCs and plasma, suggesting that it may be a biomarker associated with AF, but further validation is needed. These findings emphasize that miRNA dysregulation parallels AF burden and that certain miRNAs may serve as accessible blood-based indicators of the onset and severity of arrhythmia. This is in line with the growing evidence that miRNAs are intimately involved in AF pathophysiology and can be detected in peripheral blood, making them promising biomarker candidates for AF diagnosis and monitoring [15].

miRNAs have emerged as critical regulators in AF burden by modulating the gene expression involved in cardiac remodeling. Meanwhile, the loss of this regulatory miRNA is a key event in AF development [5,16]. Among the miRNA in our findings, *miR-1-3p* has been associated with subclinical AF, particularly in patients with cryptogenic stroke [17]. Elevated plasma levels of *miR-1-3p* have been linked to cardiac arrhythmogenesis and electrical remodeling. In addition to *miR-1-3p*, a panel of other miRNAs showed significant dysregulation in the WBCs and plasma of patients with AF. For instance, *miR-29b-3p* levels were markedly reduced in AF, while *miR-223-3p* appeared elevated, reflecting the parallel changes observed in cardiac tissue and circulation [12,18]. Previous studies have identified miR-1 as a central player in AF pathophysiology, typically reporting its downregulation in the atrial tissue of patients with AF [16,19]. Girmatsion et al. found an ~86% decrease in miR-1 levels in atrial samples from patients with PerAF, which led to the increased expression of its target Kir2.1 and contributed to AF maintenance [20]. Consistently, we observed reduced *miR-1-3p* in the circulation and WBCs of patients with AF, reinforcing the notion that miR-1 downregulation is a hallmark of AF. Our data also highlight *miR-223-3p*, which we found to be dysregulated in AF. Prior studies have established *miR-223-3p* as a pro-arrhythmic and inflammatory miRNA that is often upregulated in AF. *miR-223-3p* is known to affect cardiac ion channels (it can downregulate the potassium channel subunit KCND2, reducing the transient outward current) and promote an arrhythmogenic substrate [21]. Additionally, *miR-146a-5p* modulates inflammatory responses and has been implicated in AF initiation and progression [9], while *miR-29b* has shown antifibrotic effects by reducing atrial fibrosis and may serve as a potential therapeutic target for reversing structural remodeling in AF [22].

Other miRNAs have also demonstrated prognostic value. Both *miR-20b-5p* and *miR-330-3p* have been identified as biomarkers for atrial remodeling and arrhythmia recurrence following catheter ablation, while *miR-499-5p*, which is downregulated in AF models, has been associated with mitigating atrial fibrosis and maintaining cardiac structural integrin [23,24]. Furthermore, *miR-122-5p* has been shown to be upregulated following AF surgery and may serve as a predictor of postoperative AF recurrence [25]. Our data also indicated an upregulation of *miR-451a* in both the PaAF and PerAF groups. This aligns with a study by Lage et al., which found that circulating *miR-451a* expression is downregulated in patients with AF who experience recurrence after catheter pulmonary vein ablation, suggesting that *miR-451a* plays a role in AF recurrence by influencing fibrosis and disease progression [26]. The discrepancy between upregulation and downregulation in recurrent AF cases may be attributed to differences in the patient populations or disease stages. The experimental results demonstrated an upregulation of *miR-382-5p* in both AF groups. A study by Wang et al. [24] identified *miR-382-3p* (a closely related miRNA) as being enriched in exosomes from human pericardial fluid and associated with cardiac fibrosis [27]. While this study focused on *miR-382-3p*, it suggests that *miR-382* family members may play a role in cardiac remodeling processes relevant to AF. Further investigation is needed to confirm the specific involvement of *miR-382-5p* in AF.

The consistent change in *miR-1-3p* across all the AF groups is particularly noteworthy and may serve as a unifying molecular marker of the arrhythmia. Discrepancies between studies often stem from differences in sample types and patient populations. For instance, while *miR-1-3p* is generally found to be lower in PerAF, one study of patients with cryptogenic stroke (who later developed subclinical AF) paradoxically noted higher plasma *miR-1-3p* in those who developed AF [17]. This suggests that in an acute or pre-AF context, *miR-1-3p* levels might transiently rise, whereas in established AF, the chronic remodeling leads to the overall depletion of miR-1 in cardiac tissue and circulation. Such differences underscore how timing and disease stage can influence miRNA profiles.

The sample source is also crucial, as miRNA signatures from atrial tissue versus blood can yield different results. Some miRNAs are abundant in the myocardium but not easily detectable in plasma, and vice versa. For example, *miR-133* was significantly downregulated in animal models of AF and human atrial tissue, but its levels in the circulating blood may not always reflect that change [7,28]. Conversely, Takeshi Soeki et al. reported that *miR-328* levels are elevated in blood (particularly in the left atrial appendage plasma) of patients with AF, correlating with atrial remodeling, whereas changes in atrial tissue miR-328 were not as pronounced [29]. Our approach of analyzing both WBCs and plasma attempted to bridge this gap, and the overlap we observed (e.g., *miR-1-3p* and *miR-29b-3p* changes) confirms that some miRNAs are robust markers regardless of sample type.

Discrepancy in *miR-1-3p* expression was demonstrated in AF studies. Girmatsion et al. reported an ~86% reduction in *miR-1* in the left atrial tissue in patients with PerAF, accompanied by an increase in its target (Kir2.1) [20]. Similarly, a large cohort study in China found that plasma miR-1 levels were downregulated in patients with AF (both paroxysmal and persistent) versus healthy controls [30]. These findings align with a broad consensus that cardiac *miR-1* expression tends to be lower during AF.

In contrast, other studies have observed increased *miR-1-3p* in AF. Benito et al. found plasma *miR-1-3p* levels to be significantly higher in patients with cryptogenic stroke who developed subclinical AF (detected using implantable monitors) compared with those who remained in sinus rhythm [17]. *miR-1-3p* was the only miRNA that remained elevated upon validation and was modestly associated with AF burden. Likewise, Xiong et al. noted that patients with new-onset AF after myocardial infarction had higher circulating *miR-1* (and *miR-133*a) levels than those who did not develop AF [7]. This suggests elevated *miR-1* might precede or accompany AF onset in certain acute or paroxysmal settings. Even in experimental models, divergent patterns emerge: in a rapid-pacing animal model of PerAF, *miR-1* expression in the atrial appendage tissue was upregulated relative to the controls. These examples illustrate that *miR-1-3p* expression changes in AF are not unidirectional across all studies.

miRNAs play a significant role in modulating the molecular pathways contributing to AF, including fibrosis, inflammation, electrical remodeling, and calcium handling. In terms of fibrosis and structural remodeling, *miR-21* and *miR-29b* regulate fibroblast activation and collagen synthesis, both of which influence atrial fibrosis. Additionally, *miR-499-5p* has been shown to modulate SOX6, a factor involved in fibrotic responses in atrial tissues [8,22]. The regulation of inflammation and oxidative stress is another key mechanism underlying AF burden, with *miR-146a-5p* controlling the nuclear factor-kappa B signaling and interleukin pathways, while *miR-485-5p* regulates chemokine signaling and contributes to atrial inflammation [9].

Electrical remodeling in AF is strongly influenced by miRNAs that regulate ion channels and excitability. *miR-1-3p* and *miR-133*a-3p have been found to influence cardiac excitability, while *miR-328-3p* has been linked to calcium-handling abnormalities, thereby exacerbating the arrhythmogenic risk [28,31,32]. Table 3 illustrates the predicted target genes of *miR-1-3p* involved in AF, categorized according to the Gene Ontology (GO) terms and associated signaling pathways. Key target genes, such as *IRX-5*, *GJA1*, *PPP2R1*, *MEF2C*, *CALM2*, *SOX6*, and others, are shown with their respective annotations and involvement in pathways including gap junction signaling, calcium signaling, JAK-STAT, hypertrophic cardiomyopathy, and cAMP signaling pathways. These targets suggest the mechanistic roles that *miR-1-3p* may play in AF pathogenesis and disease progression. Metabolic regulation and autonomic nervous system impact also play an important role in AF burden. miR-181b has been implicated in endothelial–mesenchymal transition, which contributes to atrial structural changes, while lncRNA-056298, via *miR-185*, modulates autonomic nerve remodeling in AF models [33]. Additionally, *miR-223-3p* has been shown to regulate immune thrombosis, potentially linking AF to thromboembolic risk [18].

## 4. Materials and Methods

### 4.1. Study Population

**Figure 3:**
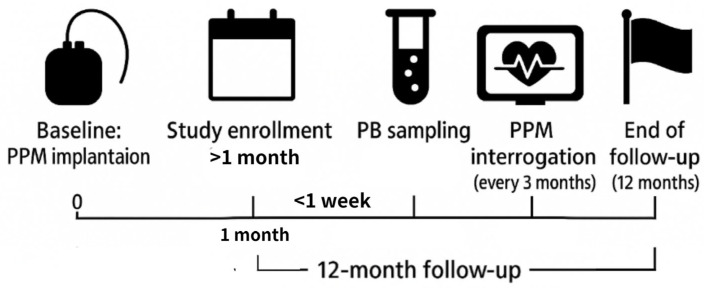
Workflow of the study design.

This study involved patients diagnosed with sick sinus syndrome who had permanent pacemaker implantation at our institute from August 2018 to December 2019. Participants with autoimmune disorders, cancers, chronic inflammation, or any documented history of AF were excluded from the control group to minimize potential undetected or subclinical AF. Patients diagnosed with sick sinus syndrome underwent permanent pacemaker (PPM) implantation between August 2018 and December 2019, and were enrolled ≥1 month after implantation to avoid acute post-implantation inflammation. A total of 73 consecutive patients were screened using PPM–Holter monitoring and classified by atrial rhythm burden into persistent AF (*n* = 15), paroxysmal AF (*n* = 28), and no AF (*n* = 30) groups. The exclusion criteria included autoimmune disease, active malignancy, or chronic inflammatory disorders. Plasma samples were pooled by age- and sex-matched groups (three pools per the no AF and PerAF arm), whereas white blood cell (WBC) samples were analyzed individually in age- and sex-matched sets without pooling. Peripheral blood was sampled within 1 week of enrollment, and PPM interrogation was performed every 3 months during a 12-month follow-up to confirm rhythm classification. This workflow is summarized in Figure 3.

Peripheral blood samples were obtained from these patients, utilizing total leukocytes and plasma for the study of miRNA expression. The research obtained clearance from the Institutional Review Board of Chang Gung Memorial Hospital (IRB number: 201702224B0) and complied with the standards established in the Declaration of Helsinki. Before the commencement of the trial, signed informed consent was secured from all the participants.

### 4.2. miRNA Isolation, Purification, and Quantification

Human blood samples were collected in EDTA blood collection tubes. Plasma and WBCs were separated using centrifugation at 1500 g for 15 min at 4 °C. Plasma samples (control: *n* = 3, PERAF: *n* = 3, each sample consisting of three plasma specimens mixed) and WBC samples (control: *n* = 2, PAF: *n* = 2, PERAF: *n* = 2) were subsequently processed for miRNA extraction. In total, three independent microarray assays were performed per group. Pooling was necessary due to the limited RNA yield from individual plasma specimens and the high cost of the array-based analysis. While technically practical, this approach may reduce the detection of inter-patient variability and must be considered a limitation of the current study. miRNAs were extracted using the miRNeasy Micro Kit (Cat #217084, Qiagen, Hilden, Germany), and the isolated miRNA was eluted with 20 μL of elution buffer. The miRNA sample quality was verified using a Nanodrop spectrophotometer (260/280 ratio > 1.8). Due to the nature of the miRNA samples, RNA Integrity Number (RIN) scores were not routinely applied; instead, a spectrophotometric assessment was used to confirm adequate purity prior to the array analysis. During the plasma sample processing, a Spike-In control (Cat. No. 219610, Qiagen, Hilden, Germany) was added as an internal reference for normalization and quality control. miRNAs isolated from both WBC and plasma were quantified using the NanoDrop™ 2000 spectrophotometer (ND-2000, Thermo Scientific, Waltham, MA, USA). RNA concentration was determined by measuring the absorbance at 260 nm (OD260). To ensure RNA quality, OD260/280 and OD260/230 ratios were also recorded to assess potential contamination from proteins or organic compounds. This step was performed using the ABI 7500 Fast Real-Time System (Applied Biosystems, Waltham, MA, USA), with PCR cycling parameters: 15 min at 95 °C, followed by 40 cycles of 15 s at 94 °C, 30 s at 55 °C, and 30 s at 70 °C.

### 4.3. miRNA Microarray Detection

The human fibrosis miScript miRNA PCR array (Cat # MIHS-117Z, Qiagen, Hilden, Germany) was used for miRNA microarray detection, analyzing 84 miRNAs that exhibit altered expression during fibrosis development. Normalization was performed using specific miRNAs included in the array (RNAU6, SNORD61, SNORD68, SNORD72, SNORD95, SNORD96A). Differential expression was identified with a fold-change of ≥1.5 or ≤0.5, *p* < 0.05, and adjusted *p*-values via the Benjamini–Hochberg correction. Additionally, unsupervised hierarchical clustering was performed on the miRNA expression data to examine similarities among samples. Euclidean distance was used as the metric for dissimilarity, and average linkage was applied for clustering. The clustering results are presented in the heatmap figures along with dendrograms, illustrating the grouping of the samples and miRNAs based on expression profiles. The data analysis was carried out using the GeneGlobe Data Analysis Center PCR software (https://geneglobe.qiagen.com/us/analyze, 18 March 2025). Differential expression was defined as >1.5-fold upregulation or <0.5-fold downregulation (relative to the controls), with statistical significance set at *p* < 0.05 [34].

### 4.4. Bioinformatic Tools

HMDD v4.0 (http://www.cuilab.cn/hmdd, 18 March 2025), miR2Disease (http://www.mir2disease.org, 18 March 2025), and miRPathDB 2.0 (https://mpd.bioinf.uni-sb.de, 18 March 2025) were used to compare and analyze the relationship between human diseases and miRNAs. GO and GO annotation analyses were performed using QuickGO (https://www.ebi.ac.uk/QuickGO/annotations, 18 March 2025), followed by the prediction of the regulatory pathways that miRNAs may be involved in using the Kyoto Encyclopedia of Genes and Genomes (KEGG).

### 4.5. Limitation

This study has several limitations. First, the small sample size (WBC: *n* = 2; plasma: *n* = 3 pooled per group) significantly limits the statistical power and generalizability. It is important to acknowledge that this study was exploratory and hypothesis-generating due to the small sample size and the specific patient cohort. The preliminary nature of our findings warrants further validation through larger-scale and longitudinal investigations. Second, pooling the plasma samples, while cost-effective, may obscure individual variability. Third, participants were limited to patients with sick sinus syndrome and pacemakers, restricting the applicability to the broader AF population. Fourth, the cross-sectional design precludes the longitudinal assessment of disease progression. Fifth, we did not measure atrial tissue miRNA levels, limiting insights into tissue-specific expression patterns. Finally, despite using Benjamini–Hochberg corrections, the risk of false-positive findings remains. Larger, longitudinal studies including healthy controls are needed to validate these exploratory findings.

## 5. Conclusions

Our study highlights *miR-1-3p* as a key miRNA associated with AF burden, demonstrating a consistent downregulation across WBCs and plasma in both patients with PAF and patients with PerAF. Additionally, several other miRNAs, including *miR-451a*, *miR-196a-5p*, and *miR-382-5p*, were identified as potential contributors to AF pathophysiology. These findings provide valuable insights into miRNA-mediated mechanisms in AF and pave the way for future biomarker development and targeted therapeutic strategies.

## Figures and Tables

**Table 1:** The common significant microRNAs’ expression analyzed using the microarray method in the WBC and plasma samples in patients with PerAF compared with control subjects.

	WBC		Plasma	
miRNA ID	PerAF	*p* Value	PerAF	*p* Value
hsa-*miR-382-5p*	2.2229		2.2670	0.04774
hsa-*miR-143-3p*	0.2837		1.7828	
hsa-*miR-338-5p*	0.6154	0.034	0.4325	
hsa-*miR-145-5p*	0.4585		0.2718	
hsa-*miR-204-5p*	2.3431		0.2051	

miRNAs included in the table with fold change compared with control subjects >1.5 or <0.5, or *p* value < 0.05.

**Table 2:** The common significant microRNAs’ expression analyzed using the microarray method in the WBC and plasma samples in patients with PaAF and patients with PerAF compared with control subjects.

	WBC		Plasma
miRNA ID	PaAF	PerAF	PerAF
hsa-*miR-196a-5p*	2.4212	2.6132	0.4246
hsa-*miR-1-3p*	0.2194	0.1995	0.2763

miRNAs included in the table with fold change compared with control subjects >1.5 or <0.5, or *p* value < 0.05.

**Table 3:** Predicted target genes and related pathways of *miR-1-3p* in AF. This figure illustrates the predicted target genes of *miR-1-3p* involved in AF, categorized according to the GO terms and associated signaling pathways. Key target genes, such as *IRX-5*, *GJA1*, *PPP2R1*, *MEF2C*, *CALM2*, *SOX6*, and others, are shown with their respective annotations and involvement in pathways including gap junction signaling, calcium signaling, JAK-STAT, hypertrophic cardiomyopathy, and cAMP signaling pathways. These targets suggest the mechanistic roles that *miR-1-3p* may play in AF pathogenesis and disease progression.

Qualifier	GO	GO Term	Symbol	Annotation	Pathway
acts_upstream_of	GO:0010880	Regulation of release of sequestered calcium ion into cytosol by sarcoplasmic reticulum	-	-	-
enables	GO:1903231	mRNA base-pairing translational repressor activity	IRX-5	Iroquois-class homeodomain protein IRX-5	-
			GJA1	Gap junction alpha-1 protein	Gap junction/Arrhythmogenic right ventricular cardiomyopathy
			PPP2R1	Serine/threonine-protein phosphatase 2A 56 kDa regulatory subunit alpha isoform	Tight junction
			MEF2C	Myocyte-specific enhancer factor 2A	Myocyte-specific enhancer factor 2A cGMP-PKG signaling pathway
			CALM2	Calmodulin-2	Calcium signaling pathway
			SOX6	Transcription factor SOX-6	-
			PIM1	Serine/threonine-protein kinase pim-1	JAK-STAT signaling pathway
			EDN1	Endothelin-1	Hypertrophic cardiomyopathy
			KCNJ2	Inward rectifier potassium channel 2	Oxytocin signaling pathway/Renin secretion
			SOX9	Transcription factor SOX-9	cAMP signaling pathway
			SPRED1	Sprouty-related, EVH1 domain-containing protein 1	MAPK signaling
			FZD7	Frizzled-7	Wnt signaling pathway
			FRS2	Fibroblast growth factor receptor substrate 2	-

## Data Availability

The data underlying this article will be disclosed to the corresponding author upon reasonable request.

## References

[1] Van Gelder I.C., Rienstra M., Bunting K.V., Casado-Arroyo R., Caso V., Crijns H., De Potter T.J.R., Dwight J., Guasti L., Hanke T. (2024). 2024 ESC Guidelines for the management of atrial fibrillation developed in collaboration with the European Association for Cardio-Thoracic Surgery (EACTS). Eur. Heart J..

[2] Vanassche T., Lauw M.N., Eikelboom J.W., Healey J.S., Hart R.G., Alings M., Avezum A., Díaz R., Hohnloser S.H., Lewis B.S. (2015). Risk of ischaemic stroke according to pattern of atrial fibrillation: Analysis of 6563 aspirin-treated patients in ACTIVE-A and AVERROES. Eur. Heart J..

[3] Abe Y., Fukunami M., Yamada T., Ohmori M., Shimonagata T., Kumagai K., Kim J., Sanada S., Hori M., Hoki N. (1997). Prediction of transition to chronic atrial fibrillation in patients with paroxysmal atrial fibrillation by signal-averaged electrocardiography: A prospective study. Circulation.

[4] Li Y., Tan W., Ye F., Wen S., Hu R., Cai X., Wang K., Wang Z. (2020). Inflammation as a risk factor for stroke in atrial fibrillation: Data from a microarray data analysis. J. Int. Med. Res..

[5] Vardas E.P., Oikonomou E., Vardas P.E., Tousoulis D. (2024). MicroRNAs as Prognostic Biomarkers for Atrial Fibrillation Recurrence After Catheter Ablation: Current Evidence and Future Directions. Biomedicines.

[6] Vardas E.P., Theofilis P., Oikonomou E., Vardas P.E., Tousoulis D. (2024). MicroRNAs in Atrial Fibrillation: Mechanisms, Vascular Implications, and Therapeutic Potential. Biomedicines.

[7] Zeng Q., Li W., Luo Z., Zhou H., Duan Z., Xiong X.L. (2023). The role of miR1 and miR133a in new-onset atrial fibrillation after acute myocardial infarction. BMC Cardiovasc. Disord..

[8] Sieweke J.T., Pfeffer T.J., Biber S., Chatterjee S., Weissenborn K., Grosse G.M., Hagemus J., Derda A.A., Berliner D., Lichtinghagen R. (2020). *miR-21* and NT-proBNP Correlate with Echocardiographic Parameters of Atrial Dysfunction and Predict Atrial Fibrillation. J. Clin. Med..

[9] Ye Q., Liu Q., Ma X., Bai S., Chen P., Zhao Y., Bai C., Liu Y., Liu K., Xin M. (2021). MicroRNA-146b-5p promotes atrial fibrosis in atrial fibrillation by repressing TIMP4. J. Cell Mol. Med..

[10] da Silva A.M., de Araújo J.N., de Freitas R.C., Silbiger V.N. (2017). Circulating MicroRNAs as Potential Biomarkers of Atrial Fibrillation. BioMed Res. Int..

[11] Glinge C., Clauss S., Boddum K., Jabbari R., Jabbari J., Risgaard B., Tomsits P., Hildebrand B., Kääb S., Wakili R. (2017). Stability of Circulating Blood-Based MicroRNAs—Pre-Analytic Methodological Considerations. PLoS ONE.

[12] Zhan J., Peng C., Liu Y., Bi Z., Lu G., Hao S., Tong Y., Zhang G. (2024). Predictive Value of Serum microRNA-29b-3p in Recurrence of Atrial Fibrillation After Radiofrequency Catheter Ablation. Clin. Interv. Aging.

[13] Xu J., Lei S., Sun S., Zhang W., Zhu F., Yang H., Xu Q., Zhang B., Li H., Zhu M. (2020). *MiR-324-3p* Regulates Fibroblast Proliferation via Targeting TGF-β1 in Atrial Fibrillation. Int. Heart J..

[14] Fattahi M., Shahrabi S., Saadatpour F., Rezaee D., Beyglu Z., Delavari S., Amrolahi A., Ahmadi S., Bagheri-Mohammadi S., Noori E. (2023). microRNA-382 as a tumor suppressor? Roles in tumorigenesis and clinical significance. Int. J. Biol. Macromol..

[15] Balan A.I., Scridon A. (2025). MicroRNAs in atrial fibrillation—Have we discovered the Holy Grail or opened a Pandora’s box?. Front. Pharmacol..

[16] Kim G.H. (2013). MicroRNA regulation of cardiac conduction and arrhythmias. Transl. Res..

[17] Benito B., García-Elías A., Ois Á., Tajes M., Vallès E., Ble M., Yáñez Bisbe L., Giralt-Steinhauer E., Rodríguez-Campello A., Cladellas Capdevila M. (2022). Plasma levels of miRNA-1-3p are associated with subclinical atrial fibrillation in patients with cryptogenic stroke. Rev. Esp. Cardiol..

[18] Dai W., Chao X., Jiang Z., Zhong G. (2021). lncRNA KCNQ1OT1 may function as a competitive endogenous RNA in atrial fibrillation by sponging miR-223-3p. Mol. Med. Rep..

[19] Chen X., Zhang Y., Meng H., Chen G., Ma Y., Li J., Liu S., Liang Z., Xie Y., Liu Y. (2024). Identification of miR-1 and miR-499 in chronic atrial fibrillation by bioinformatics analysis and experimental validation. Front. Cardiovasc. Med..

[20] Girmatsion Z., Biliczki P., Bonauer A., Wimmer-Greinecker G., Scherer M., Moritz A., Bukowska A., Goette A., Nattel S., Hohnloser S.H. (2009). Changes in microRNA-1 expression and IK1 up-regulation in human atrial fibrillation. Heart Rhythm..

[21] Zhang M.W., Shen Y.J., Shi J., Yu J.G. (2020). *MiR-223-3p* in Cardiovascular Diseases: A Biomarker and Potential Therapeutic Target. Front. Cardiovasc. Med..

[22] Han X., Wang S., Yong Z., Zhang X., Wang X. (2022). *miR-29b* ameliorates atrial fibrosis in rats with atrial fibrillation by targeting TGFβRΙ and inhibiting the activation of Smad-2/3 pathway. J. Bioenerg. Biomembr..

[23] Harada M., Okuzaki D., Yamauchi A., Ishikawa S., Nomura Y., Nishimura A., Motoike Y., Koshikawa M., Hitachi K., Tsuchida K. (2023). Circulating *miR-20b-5p* and *miR-330-3p* are novel biomarkers for progression of atrial fibrillation: Intracardiac/extracardiac plasma sample analysis by small RNA sequencing. PLoS ONE.

[24] Wang D.L., Han C., Zhao L.D., Hu G.Y., Jiang Y., Li C.G., Shi L.L., Zhou M.J. (2020). Role of miRNA-499-5p in patients with atrial fibrillation and heart failure. J. Biol. Regul. Homeost. Agents.

[25] Bai C., Liu Y., Zhao Y., Ye Q., Zhao C., Liu Y., Wang J. (2022). Circulating exosome-derived *miR-122-5p* is a novel biomarker for prediction of postoperative atrial fibrillation. J. Cardiovasc. Transl. Res..

[26] Lage R., Cebro-Márquez M., Vilar-Sánchez M.E., González-Melchor L., García-Seara J., Martínez-Sande J.L., Fernández-López X.A., Aragón-Herrera A., Martínez-Monzonís M.A., González-Juanatey J.R. (2023). Circulating *miR-451a* Expression May Predict Recurrence in Atrial Fibrillation Patients after Catheter Pulmonary Vein Ablation. Cells.

[27] Liu L., Chen Y., Shu J., Tang C.E., Jiang Y., Luo F. (2020). Identification of microRNAs enriched in exosomes in human pericardial fluid of patients with atrial fibrillation based on bioinformatic analysis. J. Thorac. Dis..

[28] Yao L., Zhou B., You L., Hu H., Xie R. (2020). LncRNA MIAT/miR-133a-3p axis regulates atrial fibrillation and atrial fibrillation-induced myocardial fibrosis. Mol. Biol. Rep..

[29] Soeki T., Matsuura T., Bando S., Tobiume T., Uematsu E., Ise T., Kusunose K., Yamaguchi K., Yagi S., Fukuda D. (2016). Relationship between local production of microRNA-328 and atrial substrate remodeling in atrial fibrillation. J. Cardiol..

[30] Lu Y., Hou S., Huang D., Luo X., Zhang J., Chen J., Xu W. (2015). Expression profile analysis of circulating microRNAs and their effects on ion channels in Chinese atrial fibrillation patients. Int. J. Clin. Exp. Med..

[31] Huang H., Chen H., Liang X., Chen X., Chen X., Chen C. (2022). Upregulated *miR-328-3p* and its high risk in atrial fibrillation: A systematic review and meta-analysis with meta-regression. Medicine.

[32] Xu R., Cui S., Chen L., Chen X.C., Ma L.L., Yang H.N., Wen F.M. (2022). Circulating miRNA-1-3p as Biomarker of Accelerated Sarcopenia in Patients Diagnosed with Chronic Heart Failure. Rev. Investig. Clin..

[33] Lai Y.J., Tsai F.C., Chang G.J., Chang S.H., Huang C.C., Chen W.J., Yeh Y.H. (2022). miR-181b targets semaphorin 3A to mediate TGF-β-induced endothelial-mesenchymal transition related to atrial fibrillation. J. Clin. Investig..

[34] Russo R., Zito F., Lampiasi N. (2021). MiRNAs Expression Profiling in Raw264.7 Macrophages after Nfatc1-Knockdown Elucidates Potential Pathways Involved in Osteoclasts Differentiation. Biology.

